# Emerging trends in management of long COVID with a focus on pulmonary rehabilitation: A review

**DOI:** 10.1111/crj.13777

**Published:** 2024-05-22

**Authors:** Allison Y. Li, Willis X. Li, Jinghong Li

**Affiliations:** ^1^ Department of Medicine University of California San Diego La Jolla California USA; ^2^ College of Engineering University of California Santa Barbara Santa Barbara California USA

**Keywords:** long COVID, pulmonary rehabilitation, telerehabilitation, wearable device

## Abstract

Long COVID, or post‐acute sequelae of COVID‐19 (PASC), represents a complex condition with persistent symptoms following SARS‐Cov‐2 infection. The symptoms include fatigue, dyspnoea, cognitive impairment, decreased quality of life in variable levels of severity. Potential mechanisms behind long COVID include vascular damage, immune dysregulation and viral persistence. Diagnosing long COVID involves medical evaluation by multidisciplinary team and assessment of persistent symptoms with scoring systems in development. Treatment strategies are symptom‐focused, encompassing multidisciplinary care, rehabilitation and tailored exercise programmes. Pulmonary rehabilitation, an effective and critical component of long COVID management, has shown promise, particularly for patients with respiratory symptoms such as dyspnoea. These programmes, which combine exercise, breathing techniques, education and psychological support, improve symptoms, quality of life and overall recovery. Innovative technologies, such as telemedicine, wearable devices, telerehabilitation, are transforming long COVID management. Telemedicine facilitates consultations and interventions, eliminating healthcare access barriers. Wearable devices enable remote and continuous monitoring of patients during their rehabilitation activities. Telerehabilitation has proven to be safe and feasible and to have high potential for COVID‐19 recovery. This review provides a concise overview of long COVID, encompassing its definition, prevalence, mechanisms, clinical manifestations, diagnosis and management approaches. It emphasizes the significance of multidisciplinary approach in diagnosis and treatment of long COVID, with focus on pulmonary rehabilitation and innovative technology advances to effectively address the management of long COVID.

## Graphical abstract

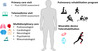

## 1 LONG COVID DEFINITION AND PREVALENCE

COVID‐19 is a respiratory viral infection caused by the severe acute respiratory syndrome coronavirus 2 (SARS‐Cov‐2). Similar to other respiratory viral infections, many patients recover within a few weeks. More often than other respiratory viral infections, some COVID‐19 patients exhibit persistent symptoms after recovering from the initial acute phase of the first 4 weeks. They have symptoms that last through the next 8 weeks of the recovery phase. They still have persistent symptoms after 12 weeks of being diagnosed with COVID‐19. Patients with such post‐COVID conditions are variously referred to as having long COVID, post‐COVID conditions, or post‐acute sequelae of COVID‐19 (PASC).^1^

Common long COVID symptoms include fatigue, shortness of breath, brain fog, muscle pain and sleep disturbances. These symptoms were first observed in late 2020, 6 months after the overwhelming COVID‐19 pandemic across the world began.^2^ The cluster of symptoms were initially found from big data analysis,^3^ then from cohort study^4^ and clinical observation.^5^, ^6^ Fair Health published a white paper on 15 June 2021.^7^ They studied longitudinal data from a database of over 34 billion private healthcare claim records, a total of 1 959 982 COVID‐19 patients, for the prevalence of post‐COVID conditions 30 days or more after their initial diagnosis. Among these patients, 23.2% had at least one post‐COVID condition. Post‐COVID conditions were found in a greater extent in patients who had more severe cases of COVID‐19, but also in patients with mild or no symptoms. Of patients who were hospitalized with COVID‐19, the percentage that had a post‐COVID condition was 50%; of patients who were symptomatic but not hospitalized, 27.5%; and of patients who were asymptomatic, 19%.^7^ Meta‐analysis showed more than 50 long‐term symptoms/effects of COVID‐19 were under the umbrella of long COVID.^8^ Most recently, a study found six clusters of long COVID patients, each with distinct profiles of phenotypic abnormalities, including clusters with distinct pulmonary, neuropsychiatric and cardiovascular abnormalities, and a cluster associated with broad, severe symptoms and increased mortality.^9^

As time went by, there were several waves of COVID‐19 infections with different SARS‐Cov‐2 variants (wildtype, Delta, Omicron etc.). The spectrum of COVID‐19 symptoms changed remarkably. It was reasonable to presume the prevalence of long COVID would change as well. Indeed, long COVID was more common in wildtype and Delta variant infections, and in those who were unvaccinated at the time of infection.^10^ After November 2021, when the Omicron variant took over, long COVID became less common. By mid‐June 2023, that percentage of long COVID had fallen from initial approximately 20% to 6%, according to CDC.^11^ In addition, a large cohort study showed that patients who were treated with Nirmatrelvir/ritonavir within 5 days of a positive SARS‐CoV‐2 test result had reduced risk of long COVID symptoms.^12^

## 2 MECHANISMS OF LONG COVID

In February 2021, 1 year after the overwhelming COVID‐19 pandemic began, NIH announced the establishment of the long COVID/PASC initiative, RECOVER Initiative, to support research that will help better understand long COVID and identify effective treatments and potential ways of preventing it.^13^ At that time, long COVID was just surfacing and we did not have much of a consensus for long COVID yet. Medical societies had to come up with temporary statements or guidelines.^14^, ^15^ Long COVID has a significant impact on individuals' quality of life and can also strain healthcare systems. Investigating the mechanisms of long COVID is needed to guide the treatment and prevention strategies. To understand the mechanism of long COVID, a large amount of research took place.^4^, ^16^ The exact mechanism of long COVID is not yet fully understood and is an area of ongoing research. Several potential mechanisms have been proposed, including microvascular and endothelial dysfunction, immune dysregulation and remnants of the SARS‐CoV‐2 virus persisting in certain tissues or organs, including the central nervous system.

COVID‐19 has been shown to affect the vascular system, including blood vessels and endothelial cells.^17^ The Spike protein damages vascular endothelium via the ACE2 receptor.^18^ Damage to these structures could lead to endothelial dysfunction, thrombosis and microvascular injuries, contributing to severe complications such as thromboembolism and multiorgan dysfunction. Studies suggest that SARS‐CoV‐2 can directly induce inflammation, oxidative stress and a procoagulant state.^19^, ^20^

Long COVID may involve an immune response that is dysregulated or prolonged, leading to ongoing inflammation and damage to various organs and systems in the body. Studies have indicated that the immune response in long COVID patients may show signs of dysregulation, with imbalances in immune cell subsets, cytokine production and immune cell function. This dysregulation can contribute to chronic inflammation and tissue damage.^21^ In addition, studies have shown that long COVID patients often have elevated levels of inflammatory markers such as IL‐6.^22^ These markers are indicative of an immune response that is not resolving as expected.

It is possible that in some cases, remnants of the SARS‐CoV‐2 virus persist in certain tissues or organs, triggering ongoing immune responses and inflammation that contribute to long‐term symptoms.^23^ Viral RNA and antigen have been detected in lung tissue post‐mortem, suggesting potential viral persistence in this organ.^24^, ^25^ Cardiac tissues have also shown evidence of viral RNA in post‐mortem studies, suggesting potential viral presence in the heart. This has raised concerns about potential cardiac involvement in long‐term COVID‐19 symptoms.^26^, ^27^ The gastrointestinal tract has been shown to be another site of SARS‐CoV‐2 infection. Studies have detected viral RNA and antigen in intestinal tissues, suggesting potential viral persistence in the gut.^28^, ^29^ Furthermore, there is emerging evidence of potential SARS‐CoV‐2 presence in central nervous system. Viral RNA has been detected in brain samples post‐mortem,^30^, ^31^ viral RNA and protein have been detected in cerebrospinal fluid,^32^ indicating viral persistence in the central nervous system. Neuroinflammation and immune response dysregulation have also been observed in post‐mortem brain samples.^33^ This is supported by reports of COVID‐19 patients experiencing symptoms like loss of taste and smell, headaches, dizziness, cognitive impairment and mood changes.^34^

## 3 CLINICAL MANIFESTATION AND DIAGNOSIS

**FIGURE 1:**
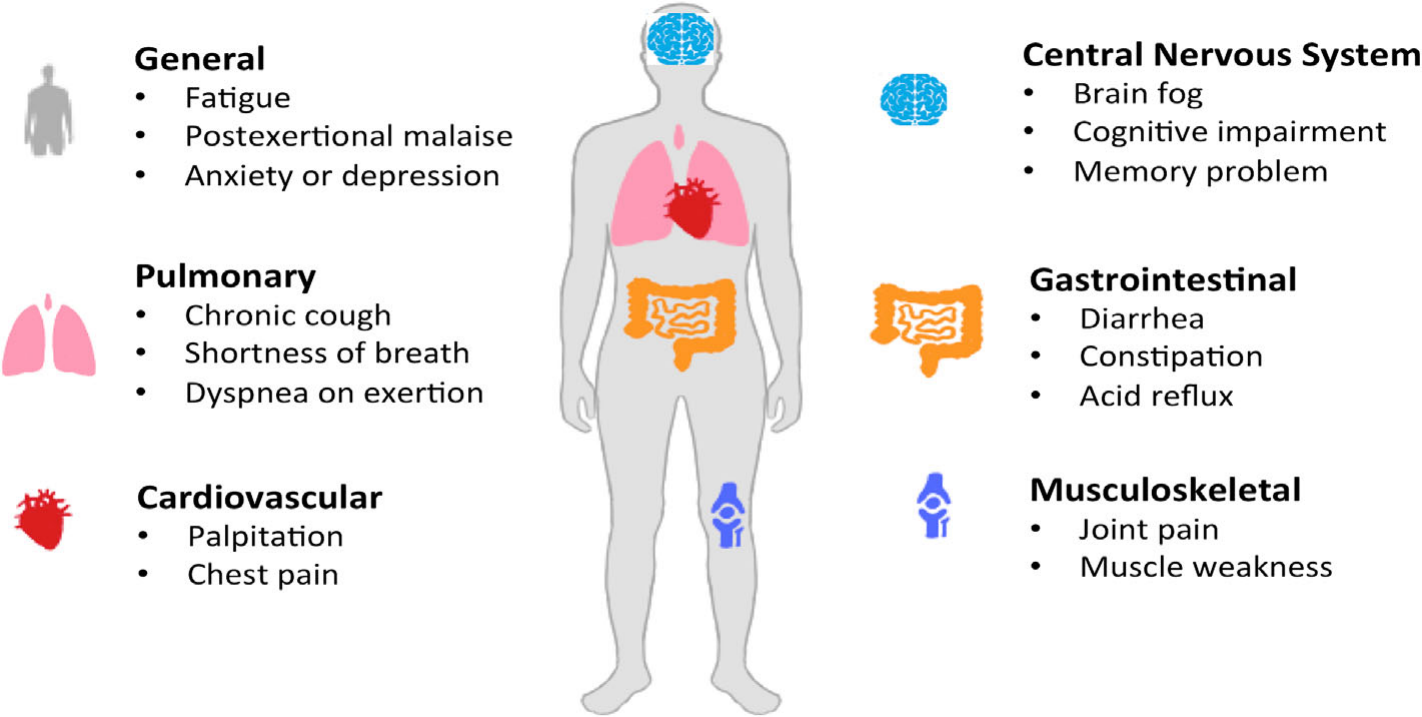
Long COVID symptoms. Fatigue or extreme tiredness, postexertional malaise, shortness of breath or difficulty breathing, brain fog or difficulty concentrating, cognitive impairment, palpitations, chest pain, muscle or joint pain, loss of taste or smell, sleep disturbances, anxiety or depression, decreased quality of life are the most common long COVID symptoms. These symptoms can persist for weeks or months after the initial SARS‐Cov‐2 infection and may fluctuate in severity.

Some common symptoms for long COVID include: fatigue or extreme tiredness, shortness of breath or difficulty breathing, brain fog or difficulty concentrating, chest pain or palpitations, muscle or joint pain, headaches, loss of taste or smell, sleep disturbances, and anxiety or depression (Figure 1). It is important to note that these symptoms can persist for weeks or even months after the initial infection and may fluctuate in severity.^35^

Diagnosing long COVID typically involves a comprehensive evaluation by healthcare professionals. There is no specific test for its diagnosis. In the long COVID clinic, a multidisciplinary team of physicians including pulmonologists, cardiologists, neurologists, psychiatrists, rheumatologists and infectious disease specialists are working together to provide comprehensive care. Blood work may be done to check for markers of inflammation, assess organ function, or rule out other medical conditions that could cause similar symptoms. Pulmonary function test (PFT) measurement including static lung volumes, expiratory flow rates and DLCO assessment are regarded as useful tools to assess long‐term lung function sequelae in patients with COVID‐19.^14^ Imaging studies, including chest X‐rays or chest CT scans may be performed to evaluate lung parenchyma.^14^ Electrocardiogram or echocardiogram tests may be done to rule out cardiac conditions. Neurological examinations or tests, such as brain imaging or nerve conduction studies, may be conducted to evaluate any potential neurological involvement.^14^

A recent study from the NIH RECOVER Initiative developed a scoring system to provide an easy way to identify long COVID patients.^10^ According to a study of nearly 10 000 Americans, researchers identified 12 ‘core symptoms’, including postexertional malaise, fatigue, brain fog, dizziness, gastrointestinal symptoms, palpitations, changes in sexual desire or capacity, loss of or change in smell or taste, thirst, chronic cough, chest pain and abnormal movements. By assigning points to each of the 12 symptoms, the team gave each patient a score based on symptom combinations. Then they identified a meaningful threshold of the score to diagnose patients with long COVID.^10^

## 4 MANAGEMENT OF LONG COVID

**FIGURE 2:**
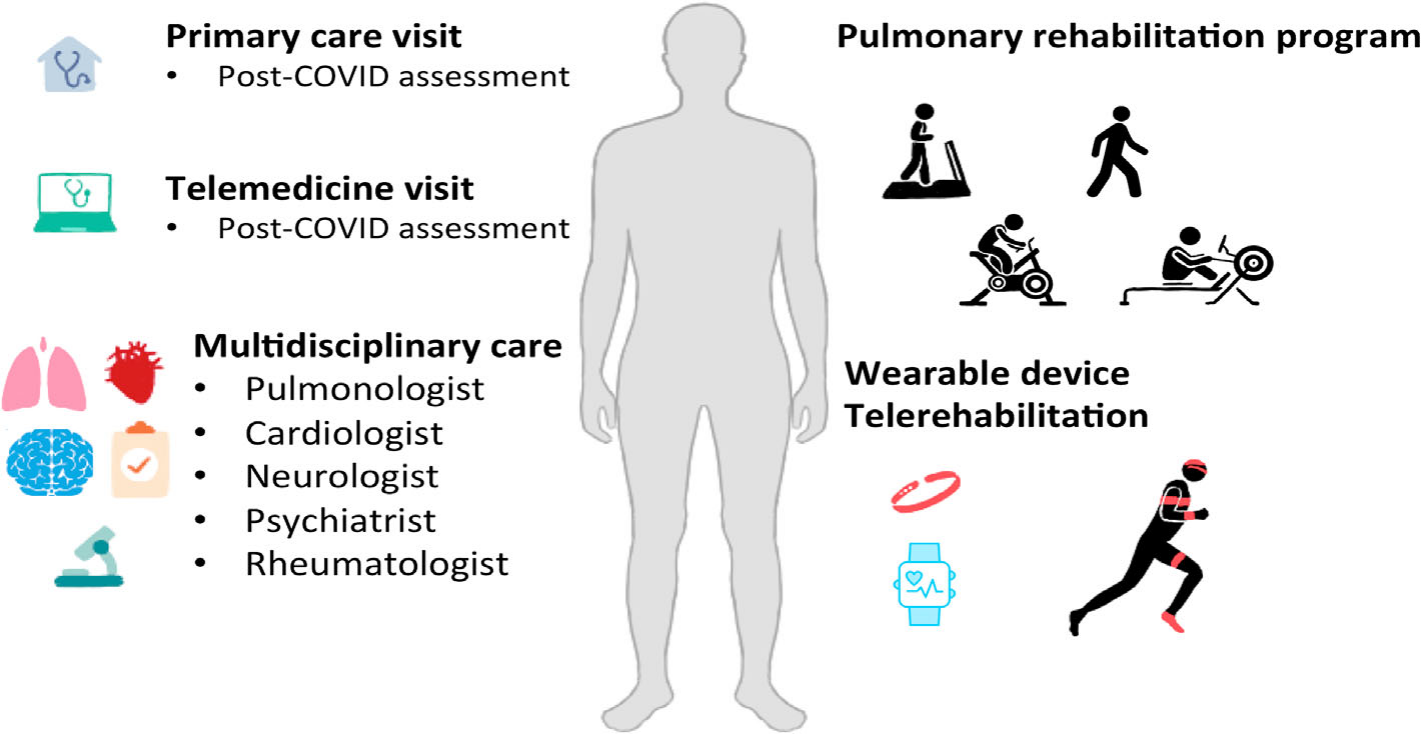
Management of long COVID. Multidisciplinary care, rehabilitation, tailored exercise programs, and psychological support are important for long COVID recovery. Pulmonary rehabilitation is an effective intervention for patients with respiratory symptoms such as dyspnoea. Innovative technologies, such as telemedicine, wearable devices, telerehabilitation, can facilitate healthcare access for long COVID‐19 patients. Telerehabilitation is proven to be safe and feasible in alleviating persistent symptoms and improving quality of life in long COVID‐19 patients.

Long COVID recovery can vary from person to person. Some individuals experience a gradual improvement over time, while others may have periods of improvement followed by relapses or fluctuations in symptom severity.^15^ One barrier to developing guidelines for long COVID is the lack of understanding of the mechanism of long COVID. In England, the National Institute for Health and Care Excellence (NICE) published a rapid guideline in December 2020 for managing the long‐term effects of COVID‐19.^36^ In the United States, as of July 2021, ‘long COVID’, also known as post‐COVID conditions, can be considered a disability under the Americans with Disabilities Act (ADA).^37^ In China, it was reported that the improvement of PFT was observed after 6 months to 1 year for severe COVID‐19 patients. Symptoms such as dyspnoea were improved over time and 6‐min walk distance test (6MWD) improved continuously.^38^ COVID‐19 survivors had longitudinal improvements in physical and mental health; however, the burden of symptomatic sequelae remained fairly high. COVID‐19 survivors had a remarkably lower health status than the general population 2 years afterwards.^39^ Systematic study of long COVID is needed to develop an evidence‐based approach for the treatment of long COVID patients.^40^ The current treatment for long COVID, focuses on managing and alleviating symptoms to improve quality of life.^41^, ^42^ There is not a single treatment that is effective for all long COVID symptoms (Figure 2). Nothing was approved by the FDA for long COVID treatment either.

Collaboration among multidisciplinary team members at the long COVID Clinic may be necessary to address specific symptoms or organ system involvement. Among these, psychological support has a significant impact in long COVID patients. Mental health support, such as counselling or therapy, can be beneficial in managing some psychological aspects. In the case of long COVID patients with neurological and cognitive issues, many of them have brain fog, poor memory, headaches, numbness and tingling in their extremities, loss of taste, or loss of smell. They may get speech therapy or occupational therapy, as in patients with cognitive decline caused by other medical conditions.^43^

The first long COVID symptomatic treatment guideline, treatment of fatigue, was issued by the American Academy of Physical Medicine and Rehabilitation.^44^ For patients with only mild fatigue who can still keep up with essential activities like work and school, activity programmes may begin with a gradual return to daily routines. As long as they have no setbacks, patients can also start with light aerobic exercise that increases in intensity and frequency over time. If symptoms do not worsen, they can ramp up exercise by about 10% every 10 days.^44^ Gradual return to activity as tolerated could be helpful for most patients. In summary, structured rehabilitation programmes may be recommended to help improve physical and cognitive function. These programmes can include exercises, breathing techniques, physical and occupational therapy, as well as neurologic rehabilitation for cognitive symptoms.^45^, ^46^ Creating a comprehensive, patient‐centred, multidisciplinary rehabilitation plan may be helpful for patients.^47^

## 5 ROLE OF PULMONARY REHABILITATION IN LONG COVID

Pulmonary rehabilitation plays a significant role in the management of chronic lung disease patients, particularly for patients experiencing respiratory symptoms and reduced lung function.^48^ Pulmonary rehabilitation programmes are designed to improve respiratory function, enhance exercise tolerance and enhance overall quality of life. These programmes typically involve a combination of exercise training, breathing exercises, education and psychological support. The exercise component may include aerobic exercises, strength training and flexibility exercises tailored to the individual's needs and capabilities. Breathing exercises can help with lung capacity, breath control and relaxation.

Pulmonary rehabilitation showed a promising positive effect on long COVID patients.^49^, ^50^ A Systematic Review and Meta‐Analysis showed that pulmonary rehabilitation could improve exercise capacity measured by 6MWD among patients with mild‐to‐moderate lung impairment after COVID‐19.^51^ In patients with long COVID, exercise capacity, functional status, dyspnoea, fatigue and quality of life improved after 6 weeks of personalized interdisciplinary pulmonary rehabilitation.^52^ Two randomized controlled trials that included 72 and 140 post‐acute COVID‐19 patients, respectively, showed that respiratory techniques have superior benefits beyond natural recovery to improve pulmonary function, exercise performance, quality of life and anxiety, especially in combination with group psychological interventions.^53^ Findings for this Year in Review revealed that exercise interventions and pulmonary rehabilitation show promise for improving functional exercise capacity, dyspnoea and fatigue in people with long COVID.^54^ Of note, the pulmonary rehabilitation programmes should be individualized and adapted to accommodate the needs of the patient as our experience accumulates.^55^

## 6 EMERGING TECHNOLOGIES IN LONG COVID MANAGEMENT

During the COVID‐19 pandemic, telemedicine experienced an unprecedented growth. Telemedicine has become more widely accepted and used by patients and healthcare providers as a means to deliver healthcare remotely, reducing the risk of virus transmission. Enhanced telemedicine platforms and apps have been developed, offering better video and audio quality, secure data transmission and user‐friendly interfaces. Moreover, wearable device technology has allowed for remote monitoring of patients' vital signs and health conditions, enabling early intervention and better disease management. Telemedicine has bridged healthcare access gaps in rural areas, where access to healthcare facilities is limited.

For COVID‐19 patients, wearable devices are designed for monitoring a set of physiological parameters that are critical for them, such as body temperature, heart rate, heart rate variability, blood oxygen saturation, respiratory rate, blood pressure and cough. This physiological information can be further used to potentially estimate lung function using artificial intelligence (AI) and sensor fusion techniques. A prototype comprises both hardware and a smartphone app has shown promising results for COVID‐19 patients and can also be used for long‐term monitoring of long COVID patients.^56^, ^57^ With the advances in technology, the general population now has the capability to continuously track vital signs, exercise output and advanced health metrics, further enhancing personal healthcare management.^58^ In this regard, wearable devices have revolutionized the personal healthcare.

The utilization of wearable devices in pulmonary rehabilitation presents an exciting frontier filled with promising opportunities. For example, these wearable devices hold the potential to enhance medication adherence. Additionally, these wearable devices are able to continuously monitor various physiological parameters of patients during their rehabilitation activities. Data can be stored on a smartphone and readily shared with healthcare providers. With this information in hand, physicians will be able to tailor medications and activity levels specifically for the individual patients. Therefore, telerehabilitation emerges as a potentially sustainable solution to address the growing burden of chronic respiratory diseases worldwide.^59^

In long COVID, fully remote telerehabilitation has proven to be safe and feasible and to have high potential for COVID‐19 recovery, offering benefits to medically complex patients including those with barriers to healthcare access.^60^ Telerehabilitation improved 6MWD, dyspnoea, performance and physical components of quality of life in long COVID patients.^61^ Incorporating remote 8‐week supervised home‐based respiratory muscle training programmes has been effective in improving quality of life in individuals with long COVID patients.^62^ The effectiveness of telerehabilitation in alleviating the symptoms of long COVID is promising.^63^ A systematic review of six articles collectively provided information on 140 patients, with variables measured including dyspnoea, fatigue, physical performance and quality of life, suggesting that the application of telerehabilitation holds great promise as an effective tool for mitigating the persistent symptoms in long COVID patients.^63^ In addition, virtual reality applications exhibit promise as an attractive and safe tool for implementing rehabilitation, enhancing performance during exercise and benefiting patients with both respiratory and cognitive symptoms.^64^ It is important to conduct higher‐quality clinical trials and systematic reviews, which are essential for providing the best available evidence on the effectiveness of telerehabilitation.^65^

The number of patients with long COVID continues to increase considerably, bringing the substantial healthcare, social and economic burdens. Current research offers many insights into the mechanisms, and possible treatments for long COVID. Pulmonary rehabilitation remains an effective and critical treatment for long COVID‐19 patients. The rise in technologies such as telemedicine and wearable devices for remote and continuous monitoring increase the healthcare accessibility for the general population, especially those who have limited access to medical care. The effectiveness of telerehabilitation in alleviating the symptoms and improving quality of life for long COVID patients is promising.

## 7 FUTURE DIRECTIONS AND CLINICAL IMPLICATIONS

As technology continues to evolve, future directions in long COVID management may involve refining wearable devices to capture a broader spectrum of physiological data, enabling more comprehensive health monitoring. Additionally, integrating advanced machine learning algorithms could enhance the predictive capabilities of wearables for personalized treatment plans. Robust clinical trials and systematic reviews are essential for standardizing and establishing the effectiveness of telerehabilitation interventions. Widespread adoption of these technologies could revolutionize post‐COVID care, providing scalable and patient‐centric approaches to address the persistent symptoms and improve the overall quality of life for individuals affected by long COVID.

## 8 CONCLUSIONS

In conclusion, long COVID represents a multifaceted challenge necessitating a comprehensive, multidisciplinary approach for effective management. The intricate interplay of vascular damage, immune dysregulation, and potential viral persistence underscores the complexity of this condition. Pulmonary rehabilitation emerges as a cornerstone in the treatment paradigm, showcasing significant efficacy, particularly in addressing respiratory symptoms. The integration of innovative technologies, including telemedicine and wearable devices, marks a transformative shift in healthcare delivery for long COVID patients. As ongoing research refines diagnostic criteria and therapeutic strategies, the future holds promise for personalized, technology‐driven interventions that enhance patient outcomes and overall quality of life in the persistent aftermath of COVID‐19.

## DATA SOURCE

The electronic database PubMed was searched using the following terms: long COVID, post‐acute sequelae of COVID‐19 (PASC), post‐COVID condition, aetiology, management, pulmonary rehabilitation, wearable device. Search filters were combined and restricted to English and human. The search was performed in September 2023.

## AUTHOR CONTRIBUTIONS

Allison Y. Li collected references, made the figures and wrote the paper. Willis X. Li collected references and wrote the paper. Jinghong Li collected references, analysed data, and wrote the paper.

## CONFLICT OF INTEREST STATEMENT

Nothing to disclose. No conflict of interest.

## ETHICS STATEMENT

No human/animal studies involved.

## Data Availability

Review article. No data generated.
